# Nutrient Inputs Alleviate Negative Effects of Early and Subsequent Flooding on Growth of *Polygonum hydropiper* With the Aid of Adventitious Roots

**DOI:** 10.3389/fpls.2022.919409

**Published:** 2022-07-22

**Authors:** Yu-Han Chen, Guan-Wen Wei, Yuan Cui, Fang-Li Luo

**Affiliations:** ^1^School of Ecology and Nature Conservation, Beijing Forestry University, Beijing, China; ^2^The Key Laboratory of Ecological Protection in the Yellow River Basin of National Forestry and Grassland Administration, Beijing, China

**Keywords:** adventitious root, early flooding, eutrophication, submergence, Three Gorges Reservoir Region

## Abstract

Riparian plants are exposed to harmful stress induced by flooding, which is often accompanied by eutrophication in the Three Gorges Reservoir Region. The phenomenon is mainly caused by domestic sewage discharges, slow water flow, and agricultural fertilizer pollution. Simulating abiotic stress, such as flooding at the initial period, can act as a signal and induce positive responses of plants to subsequent severe stress. In addition, eutrophication supplies nutrients, provides a favorable environment in the early stages of plant, and facilitates good performance in later development. However, whether early flooding (with or without eutrophication) acts as positive cue or as stress on plants at different developmental stages remains unclear. To address this question, seeds of *Polygonum hydropiper* were collected from low and high elevations in the hydro-fluctuation belt of the Three Gorges Reservoir Region. Plants germinated from these seeds were subjected to shallower and shorter early flooding treatments with or without eutrophication. Subsequently, plants were subjected to deeper and longer flooding treatments with or without eutrophication. Early flooding and eutrophic flooding significantly induced generation of adventitious roots, suggesting morphological adaptation to flooding. Although early flooding and eutrophic flooding treatments did not increase plant biomass in subsequent treatments compared with control, stem length, length and width of the 1st fully expanded leaf, and biomass of plants in the early eutrophic treatment were higher than these of the early flooding treatment plants. These results suggest a negative lag-effect of early flooding, and also indicate that nutrient inputs can alleviate such effects. Similarly, subsequent eutrophic flooding also enhanced plant growth compared with subsequent flooding, showing significantly higher values of leaf traits and adventitious root number. Plants originated from low elevation had significantly higher functional leaf length and stem biomass compared with those from high elevation. These results suggest that nutrient inputs can alleviate negative effects of early and subsequent flooding on growth of *P. hydropiper* with the generation of adventitious roots.

## Introduction

Flooding is one of the detrimental stresses for riparian plants (Ryser et al., 2011; Ayi et al., 2019). Hypoxia and low light intensity because of partial or complete coverage with water can limit photosynthesis and respiration in plants. This may result in growth retardation or even plant death (Striker et al., 2012, 2014; Kaspary et al., 2020; Kolton et al., 2020; Hartman et al., 2021). However, it has been shown that the stimulation of mild flooding at an early period can act as a signal and induce positive plant responses to subsequent severe flooding stress (Yin et al., 2014; Li et al., 2020a). When plants were subjected to flooding, both direct and indirect oxygen sensing mechanisms can quickly react to domestication responses. The resulting changes can improve the flood-tolerance of plants (Bailey-Serres et al., 2012; Yang, 2014; Herzog et al., 2016; Striker and Colmer, 2017; Striker et al., 2019). In plants, these stimulation effects of flooding are often reflected by the formation of adventitious roots within several days or even hours after flooding (Iturralde Elortegui et al., 2020; Joshi and Ginzberg, 2021). Under hypoxic stress conditions, wheat seedlings form lysigenous aerenchyma in roots and develop further, which induce advanced adaptation before the environment becomes hypoxic (Yamauchi et al., 2014; Jia et al., 2021). In addition, in response to low oxygen levels caused by shallow and prolonged flooding, plants can elongate shoots to restore contact with the atmosphere, and thus overcome flooding stress (Striker et al., 2012; Chen et al., 2019). However, negative impacts of early flooding on growth and metabolism of plant seedlings have also been shown (Lim et al., 2015; Zhang et al., 2015; Yeap et al., 2019). Whether early flooding acts as a positive induction or stress on plant seedlings and on plants at their later developmental stage remains unclear.

The Three Gorges Reservoir (TGR) in China is one of the largest reservoirs in the world (Yang et al., 2012). The water level of the TGR fluctuates repeatedly over the year from an elevation of 145 m in summer to 175 m in winter, forming a hydro-fluctuation belt (HFB) with an area of ~349 km^2^ (Lin et al., 2020). This causes plants that are naturally distributed at low and high elevations of the HFB to experience different flooding conditions and nutrient levels (Zhang et al., 2013; Liu et al., 2019). Generally, plants distributed at high elevation experience flooding at a lower frequency and for a shorter duration, while plants distributed at low elevation experience flooding at a higher frequency and for a longer duration (Schreiber et al., 2011; Chen et al., 2019). Plants at low and high elevations show different trait responses to flooding, which are particularly reflected in their leaf trait responses (Chen et al., 2009; Wei et al., 2020). Because the water level of the TGR rises gradually, early shallow flooding may induce growth responses in certain riparian species, but these responses may differ between low and high elevation plants. Moreover, in low and high elevation plants, the formation of “plastic memory” (environmentally induced phenotypic plasticity can sometimes be heritable; Portela et al., 2020) may be induced after stimulation by different levels of flooding stress for an extended period (Wei et al., 2021). This may be transmitted to the offspring through seeds produced by sexual reproduction, thus affecting the growth performance of offspring plants such as germination, biomass accumulation, and flowering. Consequently, offspring plants may adjust phenotypic strategies regulated by plastic memory to adapt to the environment that is similar to their maternal environment (Latzel et al., 2010; Alvarez et al., 2021; Jiang et al., 2021; Sánchez et al., 2021).

Eutrophication is very common in wetland ecosystems, including certain basins in the TGR region (Tercero et al., 2015; Tian et al., 2017; Huang et al., 2020; Wang et al., 2020). After the first impoundment of the TGR region in 2003, eutrophication often occurs in major tributaries (Li et al., 2020b). The effect of nutrient inputs on plant fitness at the early stage of plant development is important (Huber et al., 2012). Plant individuals that experience nourishing nutritional resources at the early stage of development often have higher phenotypic plasticity and lasting adaptability than plants that experience adverse conditions, i.e., see the silver-spoon effect (Hopwood et al., 2014). Therefore, eutrophication at an early developmental stage may alleviate flooding pressure on riparian plants (Qiu et al., 2020). In addition, the environment that parental plants experienced, or the environment offspring plants experienced early in their development may be similar to the environment adult plants will likely be exposed to. Thus, offspring plants would have higher fitness in the predicted environment at a later developmental stage (Auge et al., 2017). However, if phenotypes can respond to the parental environment and their own current environment, intragenerational plasticity is considered to evolve more easily than parental effects. The reason is that the offspring environment is more useful for predicting the future selective offspring environment than the parental environment (Alvarez et al., 2021).

The riparian plant *Polygonum hydropiper* is naturally distributed at both low and high elevations in the HFB in the TGR. This species has high phenotypic plasticity in response to flooding, and especially, plasticity in specific leaf area differs significantly between low and high elevation plants (Wei et al., 2020, 2021). Flooding significantly reduces growth, but induces adventitious root formation within hours after flooding (Wei et al., 2021). Therefore, the formation of adventitious roots can be considered as one of the most important developmental processes plants to employ when sensing flooding. In the lowest elevation (145 m) area of the TGR zone, it is difficult for plants to survive flooding, leading to low coverage and density of vegetation (Wang et al., 2009; Ye et al., 2013; Zhu et al., 2020). *P. hydropiper* is a native dominant species that naturally grows in this zone (Wang et al., 2009; Sun et al., 2011). Moreover, this species has a low demand for soil nutrients and a low nutrient release by its shoots even after extended soaking and decomposition time. Therefore, it is considered a suitable species for ecological restoration in this zone (Xiao et al., 2017). In this study, plants of *P. hydropiper* germinated from seeds collected from both low and high elevations were selected to address the following questions: (1) Does early flooding/eutrophic flooding affect the growth of plant seedlings? (2) Does early flooding/eutrophic flooding act as positive induction or stress on plant growth at a later developmental stage? and (3) Are plants from low and high elevations responding differently to early and subsequent flooding?

## Materials and Methods

### Study Material

*Polygonum hydropiper* L. (Polygonaceae) is an annual herb, which is a common and dominant species across different types of wetlands, such as rivers and lakes, in temperate Asia, Australia, Europe, and North America (Chen et al., 2019). It has branched stems, enlarged nodes, and lanceolate leaves. This species bears axillary spikelike racemes, blooms from May to September, and generates seeds from June to October (Wei et al., 2021). This species is common and distributed across all water level gradients of the TGR, and has a high percentage of importance value index in plant communities and soil seed banks (Zhang et al., 2013, 2017; Chen et al., 2020a; Zhu et al., 2020). With the water level of the TGR rises gradually, plants of *P. hydropiper* distributed at the HFB of the TGR often suffer from shallower flooding at the early developmental stage to deeper flooding at the later developmental stage (Wei et al., 2020).

### Experimental Design

Seedlings were propagated from seeds collected from three pairs of low elevation [150–155 m above sea level (a.s.l.)] and high elevation (165–175 m a.s.l.) populations in the HFB of the TGR in September 2016. The low and high elevation populations are located in Beibei District (E106°26′58.3′, N29°40′59.9′; E106°26′53.9′, N29°41′01.7′), Fuling District (E107°11′23.3′, N29°39′59.5′; E107°11′26.0′, N29°39′57.8′), and Yunyang County (E108°42′58.2′, N31°00′04.8′; E108°42′59.2′, N31°00′03.6′) in Chongqing City, China. From each population, seeds were collected from nine randomly selected plants separated by at least 2 m. All seeds were cleaned, air dried, and stored at room temperature. For the experiment, fully matured and healthy seeds with uniform size were selected from each population.

**Figure 1:**
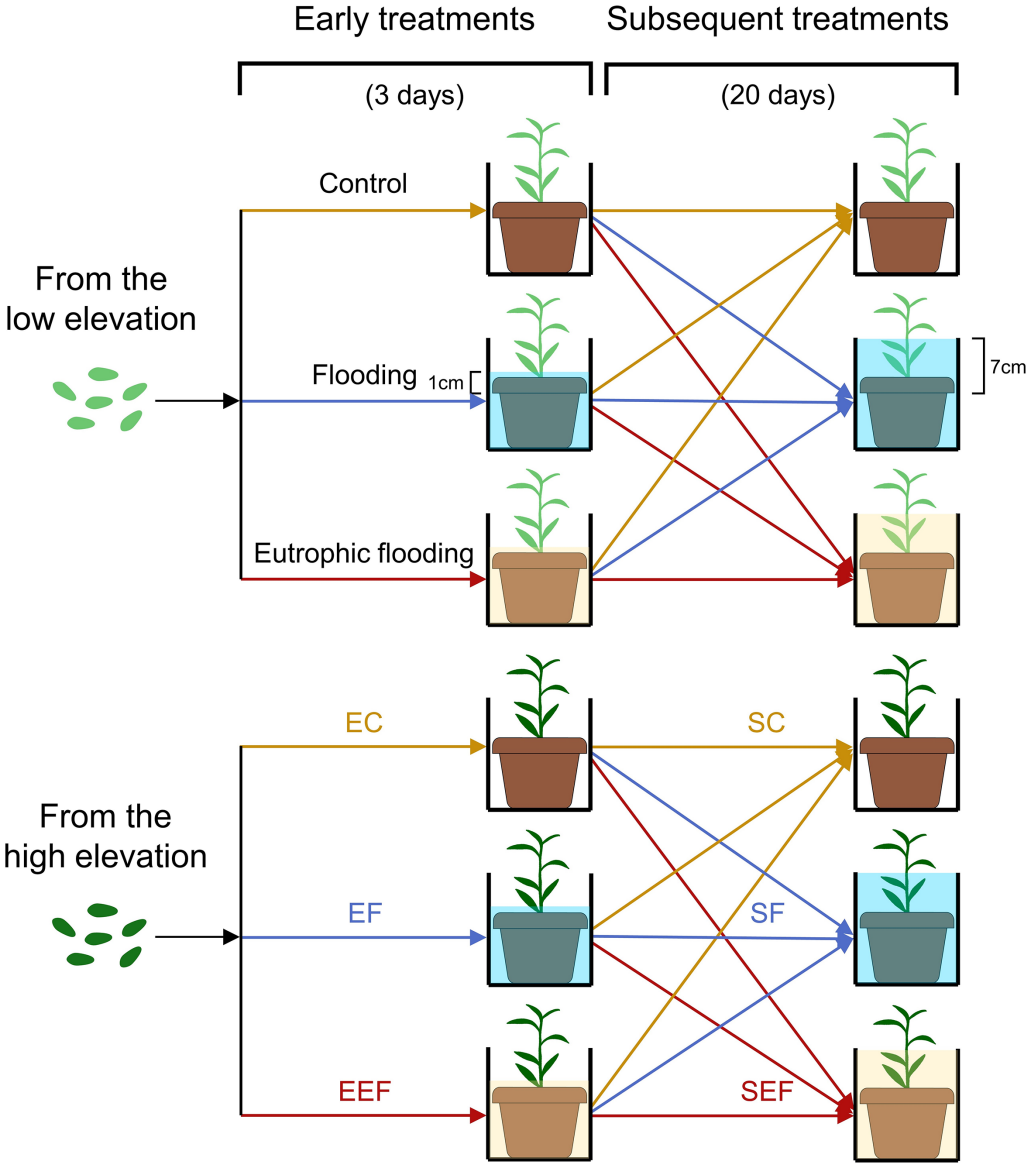
Schematic representation of the experimental design. Seeds of *Polygonum hydropiper* were collected from three populations each at low and high elevations in the Three Gorges Reservoir (TGR) region. Early treatments include early control (no flooding, EC), early flooding (using a floodwater depth of 1 cm above the soil surface, EF), and early eutrophic flooding treatments (using a eutrophic floodwater depth of 1 cm above the soil surface, EEF). After early treatments, plants were subjected to three subsequent flooding treatments: control (no flooding, SC), flooding (using a floodwater depth of 7 cm above the soil surface, SF), and eutrophic flooding (using a eutrophic floodwater depth of 7 cm above the soil surface, SEF). The N and P concentrations of eutrophic water were 2 and 0.15 mg L^−1^, respectively, which were set in reference to the eutrophication level of water in the TGR.

In June 2019, enough seeds for each population were germinated on seedling trays at the Beijing Sheng Fang greenhouse (N40°0′27.9′, E116°20′19.5′). After germination, they were transplanted into plastic pots (11 cm upper diameter, 8 cm bottom diameter, and 9.6 cm height), one seedling per pot. After most seedlings had grown to a height of 15 cm, 195 seedlings with uniform size from each population were selected for the experiment, with 1,170 seedlings in total for six populations. The substrate in pots was a quartz sand and vermiculite mixture (v:v, 1:3), containing nutrients of 1.52 ± 0.05 mg total N g^−1^ and 1.46 ± 0.07 mg total P g^−1^. In August 2019, early treatments were conducted, including three early flooding treatments × two elevations (low and high) × 65 replicates × three districts (Figure 1). The three early treatments lasted for 3 days, including early control (no flooding, keeping the soil surface slightly moist), early flooding (using a floodwater depth of 1 cm above the soil surface), and early eutrophic flooding (using a eutrophic floodwater depth of 1 cm above the soil surface). All water used in the early control and early flooding treatments was deionized water, and N and P concentrations in the early eutrophic flooding treatment were 2.0 and 0.15 mg L^−1^, respectively, which were set in reference to the eutrophication level of water in the TGR (Huang et al., 2014; Tang et al., 2015; Xiang et al., 2021). Three days later, most flooded plants (more than 95%) had generated adventitious roots. The morphologic traits of all plants were measured, including stem length, length and width of 1st fully expanded leaf (thereafter named functional leaf), total leaf number, and adventitious root number. A total of 90 plants were harvested (three early flooding treatments × two elevations × five replicates × three districts). Plants were separated into leaves, stems, and roots (both belowground roots and adventitious roots), dried, and the biomass was measured. The remaining plants that underwent early treatments were subjected to subsequent treatments resulting in three early flooding treatments × three subsequent flooding treatments × two elevations × 20 replicates × three districts. The three subsequent treatments lasted for 20 days and included: control (no flooding), flooding (where the floodwater depth was 7 cm above the soil surface, and nearly at half of the plant height), and eutrophic flooding (where the eutrophic floodwater depth was 7 cm above the soil surface). The plants subjected to subsequent treatments were subjected to three early treatments. All water used in the subsequent control and subsequent flooding treatments was deionized water, the N and P concentrations in the eutrophic flooding treatment were 2.0 and 0.15 mg L^−1^, respectively. Starting at the seed germination, the experiment lasted for 3 months. The air temperature in the greenhouse ranged from 28°C to 35°C and the relative air humidity ranged from 40% to 60% at noon.

### Growth Measurements

The time of harvesting of these 90 plants after 3 days of induction treatments was defined as day 0 of subsequent treatments. During subsequent treatments, the adventitious root number was measured on days 1, 3, 5, 7, 10, 13, 16, and 20. A total of 270 plants (three early flooding treatments × two elevations × five replicates × three subsequent flooding treatments × three districts) were harvested every 5 days for four times and dried in an oven at 70°C for at least 72 h to measure root biomass on days 5, 10, and 15, and leaf, stem, root, and adventitious root biomass on day 20. Then, the root to shoot ratio was calculated.

### Data Analyses

Before analyses, data were assessed for normality and homogeneity of variance. For early treatments, two-way ANOVA was used to test for effects of early treatments (fixed effect) and elevation (fixed effect) on stem length, functional leaf length and width, total leaf number, leaf biomass, stem biomass, root biomass, total biomass, root to shoot ratio, and adventitious root number. For subsequent treatments, three-way ANOVA was used to test for effects of early treatments (fixed effect), subsequent treatments (fixed effect), and elevation (fixed effect) on stem length, functional leaf length and width, total leaf number, leaf biomass, stem biomass, root biomass, total biomass, root to shoot ratio, adventitious root number, and adventitious root biomass. Plant height on day 1 of the subsequent treatments was set as covariate for all growth variables. Analyses were conducted using R, version 4.1.1 (R Core Team, 2021).

## Results

### Effects of Early Treatments on Plant Performance

**Figure 2:**
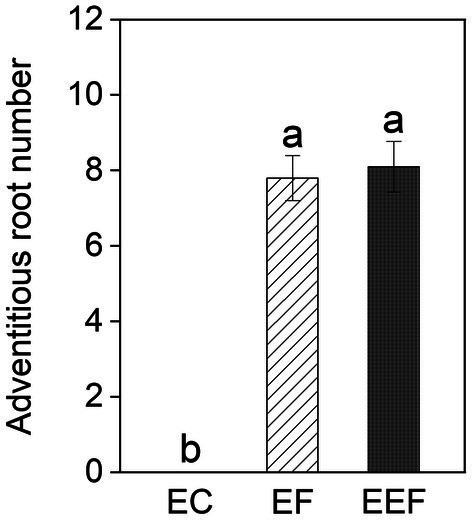
Adventitious root number of *P. hydropiper* subjected to early control (EC), early flooding (EF), and early eutrophic flooding (EEF) at the end of early treatments. Different lowercase letters represent significant differences among the three early treatments.

Early treatments had significant effects on adventitious root number but did not significantly affect other growth variables (Table 1). The generation of adventitious roots in both early flooding and eutrophic flooding treatments started on day 1 and retained a high generation rate until day 7, but no adventitious roots were found in early control treatment (Supplementary Figures 1A–C). Early flooding and eutrophic flooding treatments did not show significant differences in adventitious root number (Figure 2). The elevation and interaction of elevation and early treatments had no significant effect on any growth variable (Table 1).

**Table 1:** ANOVA results for effects of early treatments (ET: early control, early flooding, and early eutrophic flooding) and elevations (E: low and high) on stem length, functional leaf length and width, total leaf number, leaf biomass, stem biomass, root biomass, total biomass, root to shoot ratio, and adventitious root number in *Polygonum hydropiper* at the end of early treatments.

Trait	Early treatments		Elevation		ET × E	
	(ET)		(E)			
	*F*_2,84_	*p*	*F*_1,84_	*p*	*F*_2,84_	*p*
Stem length	0.06	0.944	0.01	0.909	0.42	0.660
Functional leaf length	0.20	0.818	1.10	0.297	0.48	0.623
Functional leaf width	0.91	0.409	0.35	0.556	2.59	0.081
Total leaf number	0.09	0.919	1.33	0.252	1.95	0.149
Leaf biomass	0.04	0.957	0.45	0.504	2.12	0.126
Stem biomass	0.12	0.886	3.30	0.073	1.61	0.206
Root biomass	1.30	0.279	1.15	0.287	1.76	0.179
Total biomass	0.07	0.929	1.50	0.223	1.98	0.145
Root to shoot ratio	1.35	0.263	1.71	0.195	0.23	0.798
Adventitious root number	76.95	**<0.001**	0.85	0.361	0.24	0.790

A *p*-value smaller than 0.05 is formatted in bold.

**Figure 3:**
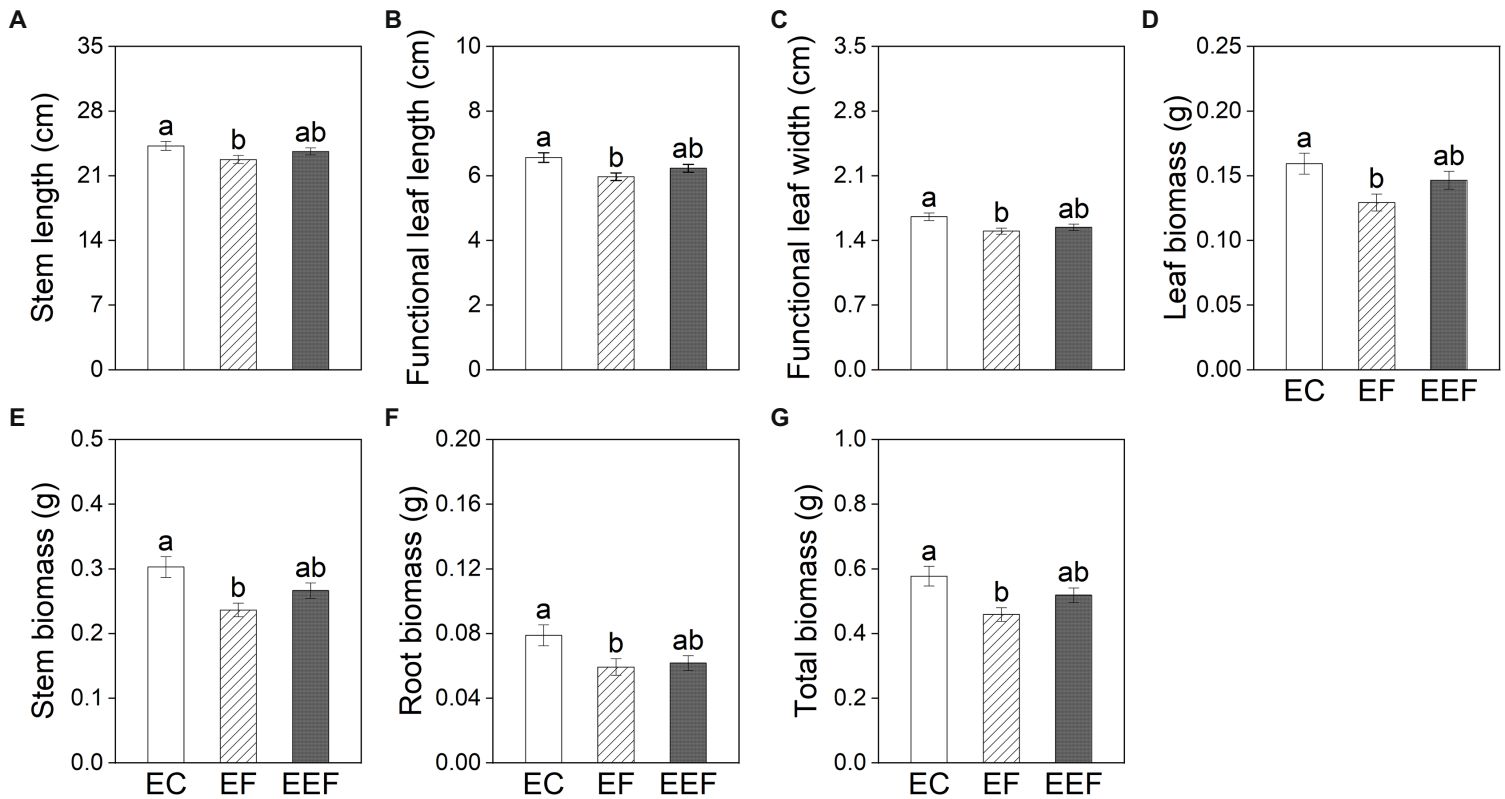
Stem length **(A)**, functional leaf length **(B)** and width **(C)**, leaf biomass **(D)**, stem biomass **(E)**, root biomass **(F)**, and total biomass **(G)** of *P. hydropiper* subjected early control (EC), early flooding (EF), and early eutrophic flooding (EEF) at the end of subsequent treatments on day 20. Different lowercase letters represent significant differences among early treatments.

Early treatments had significant effects on all growth variables except for root to shoot ratio and adventitious root biomass of plants at the end of subsequent treatments (Table 2). The stem length, functional leaf length and width, leaf biomass, stem biomass, root biomass, and total biomass of plants that had been subjected to early control treatment showed no significant differences compared with early eutrophic treatment, but were significantly higher than early flooding treatment (Figure 3).

**Table 2:** ANOVA results for effects of early treatments (ET: early control, early flooding, and early eutrophic flooding), subsequent treatments (ST: control, flooding, and eutrophic flooding), and elevation (E: low and high) on stem length, functional leaf length and width, total leaf number, leaf biomass, stem biomass, root biomass, total biomass, root to shoot ratio, adventitious root number, and adventitious root biomass in *P. hydropiper* at the end of subsequent treatments on day 20.

Trait	Early treatments (ET)	Subsequent treatments (ST)	Elevation (E)	ET × ST	ET × E	ST × E	ET × ST × E
	*F*_2,251_	*F*_2,251_	*F*_1,251_	*F*_4,251_	*F*_2,251_	*F*_2,251_	*F*_4,251_
Stem length	24.38^***^	0.65	0.16	0.41	0.29	0.17	1.43
FLL	16.22^***^	1.26	22.69^***^	1.95	0.38	0.00	0.10
FLW	8.93^***^	4.35^*^	4.86^*^	0.32	1.21	0.12	1.33
Total leaf number	3.36^*^	30.77^***^	0.55	1.02	0.75	0.02	1.34
Leaf biomass	13.58^***^	6.78^**^	2.72	1.86	2.31	0.08	1.13
Stem biomass	29.02^***^	2.33	13.19^***^	1.22	1.62	0.07	0.60
Root biomass	20.63^***^	147.87^***^	1.56	1.66	1.30	0.43	0.92
Total biomass	23.22^***^	3.49^*^	7.29^**^	1.75	2.12	0.05	0.84
Root to shoot ratio	0.55	4.95^**^	0.94	1.35	1.80	0.24	0.29
ARN	4.55^*^	501.68^***^	1.96	1.24	0.53	0.31	0.34
ARB	1.69	101.50^***^	0.24	1.10	0.62	0.29	0.40

FLL, functional leaf length; FLW, functional leaf width; ARN, adventitious root number; ARB, adventitious root biomass. *p*-values smaller than 0.05 are formatted in bold. No symbols *p* > 0.05.

*** *p* < 0.001;

** *p* < 0.01;

* *p* < 0.05.

### Effects of Subsequent Treatments on Plant Performance

**Figure 4:**
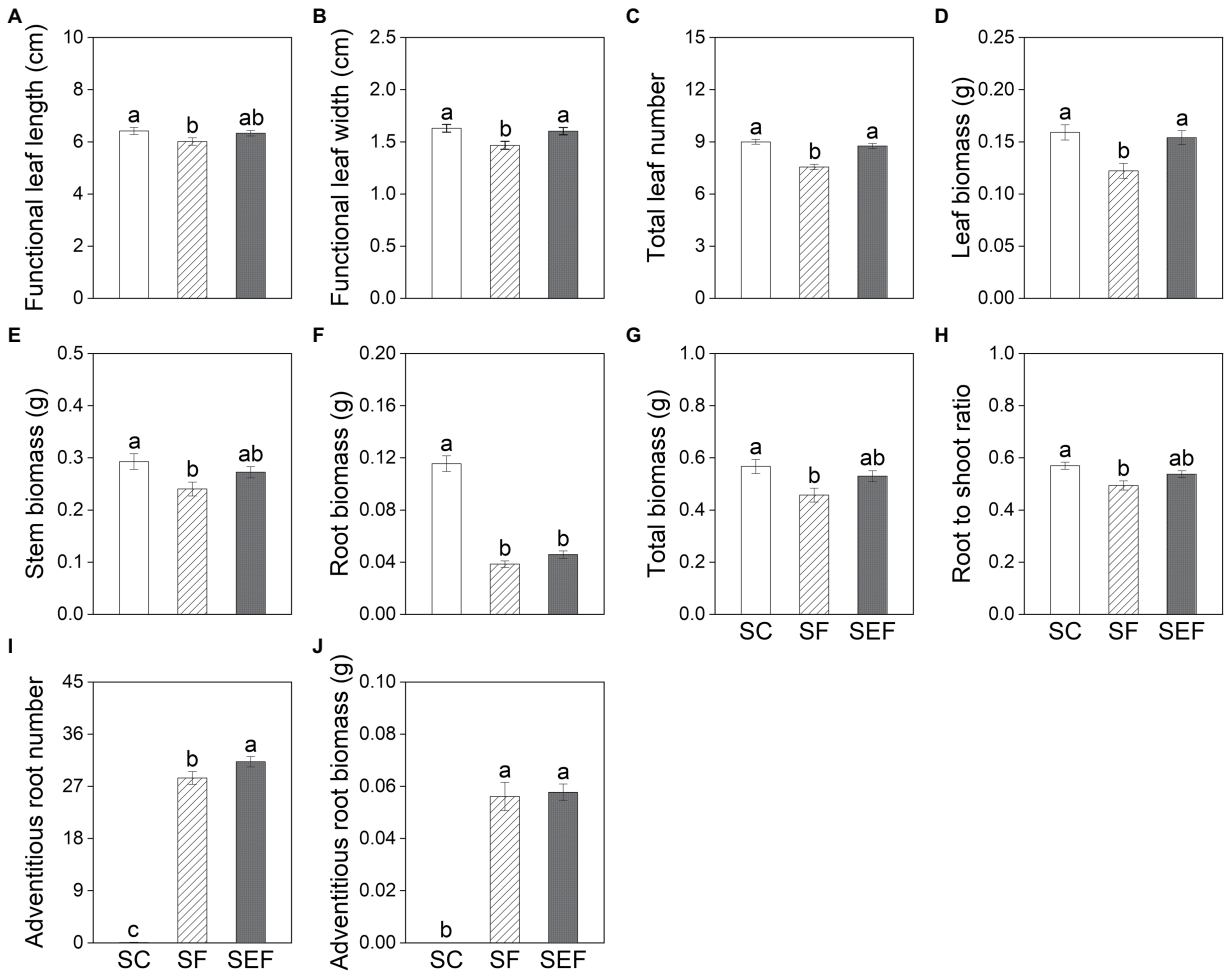
Functional leaf length **(A)** and width **(B)**, total leaf number **(C)**, leaf biomass **(D)**, stem biomass **(E)**, root biomass **(F)**, total biomass **(G)**, root to shoot ratio **(H)**, adventitious root number **(I)** and biomass **(J)** of *P. hydropiper* subjected to subsequent treatments of control (SC), flooding (SF), and eutrophic flooding (SEF) at the end of subsequent treatments on day 20. Different lowercase letters represent significant differences among subsequent treatments.

**Figure 5:**
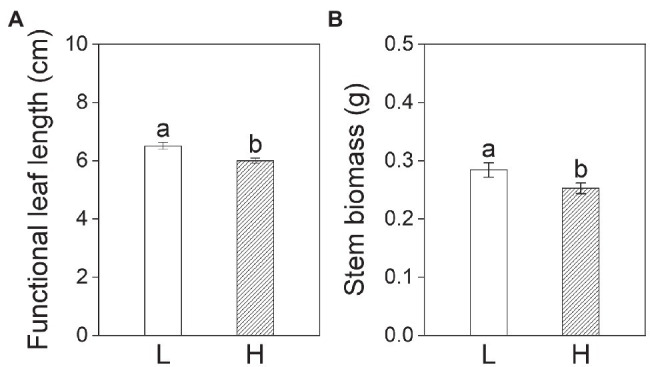
Functional leaf length **(A)** and stem biomass **(B)** of *P. hydropiper* from low (L) and high (H) elevations on day 20. Different lowercase letters represent significant differences between two elevations.

Subsequent treatments had significant effects on all growth variables except for stem length, functional leaf length, and stem biomass of plants on day 20 (Table 2). The leaf width, leaf number, and leaf biomass of plants in the subsequent control and eutrophic flooding treatments were significantly higher than in the subsequent flooding treatment (Figures 4B–D). The leaf length, stem biomass, total biomass, and root to shoot ratio of plants in the subsequent control treatment were not significantly different compared with subsequent eutrophic treatment. However, these variables were significantly higher than in subsequent flooding treatment (Figures 4A,E,G,H). Both subsequent flooding and eutrophic flooding treatments stimulated the generation of adventitious roots, with a higher number in subsequent eutrophic flooding; however, these two treatments significantly suppressed root growth (Figures 4F,I,J; Supplementary Figures 1D–F). Elevation had significant effects on functional leaf length and width, stem biomass, and total biomass (Table 2). Plants that originated from the low elevation had significantly higher functional leaf length and stem biomass compared with plants from the high elevation (Figure 5).

## Discussion

### Growth Responses of *Polygonum hydropiper* to Early Flooding Treatments

The generation of adventitious roots appeared quicker than other changes in shoot traits during early flooding treatments (Panozzo et al., 2019; Joshi and Ginzberg, 2021). Early flooding and eutrophic flooding treatments quickly generated adventitious roots but did not affect other growth variables (Supplementary Figure 1). Fast generation of adventitious roots may facilitate oxygen diffusion and improve aeration of flooded organs (Ayi et al., 2016; Park et al., 2020), which has been proved to be caused by ethylene accumulating because of limited gas diffusion in floodwater (Voesenek and Bailey-Serres, 2015; Herzog et al., 2016; Joshi and Ginzberg, 2021). In response to flooding, plants often prioritize activities related to survive such as aeration and nutrient absorption, with faster responses in roots than in shoots (Striker et al., 2014; Vidoz et al., 2016; Kaspary et al., 2020).

### Effects of Early Treatments on Growth of *Polygonum hydropiper* at Later Developmental Stage

Studies have shown that when plants experience abiotic stresses at the early developmental stage, this experience can stimulate responses enabling plants to better cope with subsequently experienced severer stress (Harb et al., 2010; Hatzig et al., 2018; Nosalewicz et al., 2018; Avramova, 2019; Mantoan et al., 2020; Li et al., 2020a, 2022; Zamorano et al., 2021). Some studies have also found no significant effects that early stresses have on plant performance (Ploschuk et al., 2020; Tang et al., 2022). The results of the present study showed that short early flooding induced morphological adaptation to flooding, manifesting as the generation of adventitious roots. However, it did not improve growth responses to subsequent flooding, and a significant decrease in growth variables was observed. In comparison, plants that had experienced early eutrophic flooding treatment did not show a significant decrease in growth variables compared with plants in the control treatment at the end of subsequent treatments. These results suggest negative lag effects of early flooding, further indicating that nutrient inputs can alleviate such effects.

Flooding has detrimental effects on the growth of many riparian plants; however, under conditions of nutrient enrichment, plants can absorb and utilize nutrients to offset the negative effects of flooding (Qiu et al., 2020). Plant individuals growing in favorable habitats (e.g., with high nutrient availability) at the early seedling stage often have higher fitness at their adult stage (Engqvist and Reinhold, 2016). Especially, when the conditions at later developmental stages are comparatively hostile, such an effect becomes more important (Portela et al., 2020). Favorable resource conditions such as light and nutrient availability at the early stage also determine the subsequent phenotypic responses of *Rumex palustris* and may produce different adaptive strategies in response to spatially or temporally heterogeneous conditions (Chen et al., 2011; Huber et al., 2012). Early eutrophic conditions were beneficial for plants in coping with flooding but did not significantly increase plant growth compared with control at the end of subsequent treatments. The possible reason could be that the positive effects of nutrient inputs are not sufficiently strong to offset negative effects of flooding, resulting in a negative effect overall (Nguyen et al., 2018b). Alternatively, several studies on the silver spoon effects have found a time-lag effect (Germain and Gilbert, 2014; van Allen et al., 2021; Luo et al., 2022).

Subsequent eutrophic flooding also improved plant growth compared with subsequent flooding, resulting in significantly higher growth of leaves and adventitious roots (Figure 4). These results indicate a higher adaptation of plants to subsequent eutrophic flooding, suggesting that the effects of nutrient inputs are beneficial for plant flood-tolerance (Joshi and Ginzberg, 2021). In this study, nitrate was added which reportedly plays a beneficial role in maintaining photosynthetic metabolism during short-term flooding (Nguyen et al., 2018a; Borella et al., 2019; Posso et al., 2020; Da-Silva et al., 2021). Upon flooding, because of the inhibited growth of belowground roots, plants of *P. hydropiper* may prioritize the allocation of resources obtained from eutrophic water to expand leaf lamina, and elongate leaves and adventitious roots.

### Different Responses of *Polygonum hydropiper* Plants Originated From Low and High Elevations

The compensation for biomass loss of offspring plants under stress is not only driven by the environment offspring plants experience, but it can also be driven by their parents (Latzel et al., 2010; Quan et al., 2021). The environmental stress experienced by parental plants can form a kind of “memory,” which affects seeds and the growth performance of offspring plants (Bossdorf et al., 2009; Li et al., 2021; Waterman and Sultan, 2021). Compared with plants from high elevation, plants from low elevation had significantly higher stem biomass and functional leaf length. For the maternal generation, compared to populations from the low elevation, *P. hydropiper* populations that originated from the high elevation are often flooded less frequently and covering water is shallower. The maternal experience of high-elevation plants may weaken the ability of their offspring plants to respond to sudden flooding stress. In contrast, offspring plants that originated from the low elevation may have higher adaptability in response to this “predictable” periodic stress (Herman et al., 2012; van Dooren et al., 2020). Our former studies also reported such different strategies of *P. hydropiper* populations in low and high elevations (Chen et al., 2020b; Wei et al., 2020, 2021). The differences in flooding regimes of low and high elevations may also induce epigenetic variation in the maternal generation, which can be transmitted to the offspring, leading to phenotypic variations (González et al., 2016, 2018; Benson et al., 2021; Sánchez et al., 2021).

## Conclusion

The results clearly showed that early flooding and eutrophic flooding treatments quickly induced the generation of adventitious roots in *P. hydropiper*. Early flooding exerted a negative lag-effect on plant growth at the end of subsequent flooding, which could be alleviated by nutrient inputs. Moreover, nutrient inputs also alleviated negative effects of subsequent flooding. Therefore, nutrient inputs can alleviate negative effects of early and subsequent flooding because of the quick induction of new adventitious roots. However, if plants do not generate adventitious roots upon flooding, the effect of nutrient inputs may not be apparent, which will be further studied in the future. Furthermore, the growth of riparian flood-tolerant species could be promoted under eutrophic conditions in the reservoir area.

## Data Availability Statement

The raw data supporting the conclusions of this article will be made available by the authors, without undue reservation.

## Author Contributions

Y-HC, G-WW, and F-LL conceived and designed the study. G-WW participated in the field work and provided study materials. Y-HC carried out greenhouse experiments, analyzed the data, and wrote the manuscript. YC and F-LL participated in the structuring and editing of the manuscript. F-LL wrote and revised the manuscript. All authors contributed to the article and approved the submitted version.

## Funding

This work was supported by the Fundamental Research Funds for the Central Universities (grant number 2021ZY90) and the National Natural Science Foundation of China (grant number 32071525).

## Conflict of Interest

The authors declare that the research was conducted in the absence of any commercial or financial relationships that could be construed as a potential conflict of interest.

## Publisher’s Note

All claims expressed in this article are solely those of the authors and do not necessarily represent those of their affiliated organizations, or those of the publisher, the editors and the reviewers. Any product that may be evaluated in this article, or claim that may be made by its manufacturer, is not guaranteed or endorsed by the publisher.
