# Supplementary material for: Nutrient Inputs Alleviate Negative Effects of Early and Subsequent Flooding on Growth of Polygonum hydropiper With the Aid of Adventitious Roots
Source: Front Plant Sci. 2022 Jul 22;13:919409. doi: 10.3389/fpls.2022.919409 (PMC9355131; doi:10.3389/fpls.2022.919409)
Supplement: Supplementary file 1 [file Data_Sheet_1.DOCX]

Supplementary Material

# Supplementary Figure

**Supplementary Figure 1** Adventitious root number and root biomass of *Polygonum hydropiper* at different dates under the subsequent treatments of control (SC), flooding (SF), and eutrophic flooding (SEF) after early control, early flooding, and early eutrophic flooding treatments. Symbols indicate significant differences among treatments at each date. Symbols: ^***^, *p* < 0.001; ^**^, *p* < 0.01; ^*^, *p* < 0.05, and no symbols *p* ≥ 0.1.
